# KNOWLEDGE, ATTITUDE AND RISK PERCEPTION OF COVID-19 AMONG NIGERIANS

**DOI:** 10.21010/Ajid.v16i2.7

**Published:** 2022-05-06

**Authors:** Fadeyi Abayomi, Adeoti Adekunle Olatayo, Adeboye Muhammed Akanbi Nurudeen, Awosanya Joseph Abioye, Oluwadiya Ibironke Omowumi, Oluwadiya Kehinde Sunday

**Affiliations:** 1 Medical Microbiology & Parasitology Department, University of Ilorin, Ilorin, Kwara State, Nigeria; 2 Internal Medicine Department, Ekiti State University, Ado-Ekiti, Ekiti State, Nigeria; 3 Paediatric & Child Health Department, University of Ilorin, Ilorin, Kwara State, Nigeria; 4 Medical Microbiology &Parasitology Department, Ekiti State University, Ado-Ekiti, Ekiti State, Nigeria; 5 Demography & Social Statistics Department, Obafemi Awolowo University, Ile Ife, Osun State, Nigeria; 6 Orthopedic Unit, Surgery Department, Ekiti State University, Ado-Ekiti, Ekiti State, Nigeria

**Keywords:** COVID-19, Pandemics, Risk, Knowledge, Attitude Perception, Nigeria

## Abstract

**Background:**

COVID-19 emerged as a novel disease with global health importance. Personal and collective behaviours have been modified to prevent the spread of this pandemic. This study is aimed to assess the knowledge, attitude and risk perceptions of Nigerians towards COVID-19.

**Method:**

A cross-sectional survey was conducted between the 30th of April and 17th of May 2020 with a national representative among Nigerians using a combination of online and interviewer administered questionnaire.

**Results:**

A total of 1,135 respondents participated in the study with a slight male preponderance (M: F=1.5:1). The mean age of the participants was 42±12.2 years with the majority (77%) between the ages of 26 and 55 years. The majority were aware of the pandemic, mostly through mass and social media. Most of the respondents demonstrated good knowledge of COVID-19 but the unaware minority (0.6%) were likewise uneducated. Educational status had no significant association with attitude towards hand washing and wearing of face mask (p>0.05). Risk perception was however low to moderate despite their concerns for COVID-19 and lack of trust in the governments’ response to the disease.

**Conclusion:**

The risk perception of COVID-19 among the respondents is not encouraging, hence more advocacy and public orientation must be done to curb further spread of COVID-19 in our setting.

## Introduction

In late December 2019, the World Health Organization (WHO) reported the emergence of a viral pneumonia among patients in Wuhan city, China (Kebede *et al.*, 2020). This was subsequently named COVID-19, a pan-systemic disease caused by a novel human coronavirus; severe acute respiratory syndrome-corona virus (SARS-COV-2) and it has become a pandemic (Jin *et al.*, 2020).The incubation period of SARS-COV-2 virus is 2–14 days with most patients being asymptomatic or mildly symptomatic (Campus *et al.*, 2020; Kebede *et al.*, 2020).

In Nigeria, the first case of COVID-19 was recorded on February 27, 2020 in Lagos and there are 255,190 recorded cases as at March 23, 2022 .(*NCDC Coronavirus COVID-19 Microsite*). With the current high global disease burden and associated fatalities, the management of the pandemic primarily depends on strict adherence to the recommended precautionary measures, and the administration of vaccine as well as recently approved medications for the treatment of COVID-19 patients. The effectiveness of these measures is mostly affected by the knowledge, attitudes, and risk perception of the public. Ultimately, the individual’s knowledge, attitude and risk perception are crucial to influencing decision related to risky behaviours, as individuals with lower risk perception tend to reduce preventive behaviours, while people with high-risk perception tend to take preventive behaviour (Ding *et al.*, 2020).

Effective control of this pandemic would, therefore, require an understanding of risk perceptions of individuals’ willingness, motivation and capacity to adopt the preventive strategies which influence their engagement and social interaction within the community (Seale *et al.*, 2020). Furthermore, the importance of risk management and effective risk communication by the expert cannot be overemphasised during a pandemic of this magnitude (Cori *et al.*, 2020). It is imperative that risk perception researches on COVID-19 be conducted to improve health risk communication, build trust, and improve collaborating governance and policy development (Cori *et al.*, 2020; Ding *et al.*, 2020).

The projections on risk information about COVID-19 is characterised by uncertainty and has been very dynamic over the past months. It is unclear how Nigerians perceive the risks or whether their risk perceptions influenced their decisions about protective actions. A questionnaire survey on the risk perception of COVID-19 among adult Nigerians would have great significance for the control of the epidemic in Nigeria and other countries in sub-Saharan Africa. The purpose of the study was to evaluate the risk perception of COVID-19 among adult Nigerians.

## Materials and Methods

This is a combination of an online and interviewer-administered questionnaire survey of the risk perception of COVID-19 by Nigerians.

We calculated a quota sample of 1066 participants. We based this on a variance of population of 50%, Confidence Level of 95%, a margin of error of 3 and infinite population size (Taherdoost, 2017).

We created the online questionnaire with Google Forms, a free open-source software survey tool available on the internet. On April 30, 2020, we recruited the respondents throughout Nigeria with quota sampling. We sent the link to the forms to individuals and through WhatsApp and Telegram groups. The invitation included a detailed description of the study. Respondents were informed of their confidentiality and freedom to withdraw from the survey at any stage when so desired. The study was approved by the Ethical Review Committee of Ekiti State University Teaching Hospital, Ado-Ekiti, Ekiti State, Nigeria. The questionnaire required approximately 10 to 15 minutes to complete; it needed to be brief to maximise the response rate (The iConnect consortium *et al.*, 2011). We sent to all groups and individuals who did not respond, reminders two times a week until the survey closed on May 17, 2020.

In addition, a trained interviewer administered the questionnaire to consenting participants in Ile-Ife in Southwest Nigeria. A convenience sampling technique of the participants in the farm settlements in Ile-Ife was used for the purpose to capture those who are not on social media. We gave cloth face masks to the respondents to wear, and we observed social distancing during the interviews to prevent the transmission of infection between the asymptomatic individuals and the interviewer.

We downloaded the results as Microsoft Excel document and imported it into IBM-SPSS Version 25 for analysis. We grouped educational status into three. Those with primary, secondary and no formal education were grouped into lower educational status. Those who are still in higher institutions were grouped into middle educational status and graduates were grouped into highest educational status. We used this grouping to analyse interaction between level of education and some selected variables using chi-square statistics. The questionnaire and the responses that applied in this study are included as supplementary material in the tables and the Appendix.

Ethical approval was obtained from the research review board of Ekiti State University Teaching Hospital, Ado-Ekiti, Nigeria with protocol number-EKSUTH/A67/2020/10/002. The information obtained was made anonymous and coded prior to analysis to ensure confidentiality.

## Results

### Demographics

We received 1135 responses comprising 423 (38.8%) females and 667 (61.2%) males (Table 1). Forty-five respondents didn’t give their gender on the questionnaire. The mean age was 42±12.2 years. Seventy-nine percent of the respondents fall within the 26-55year age bracket. Seventy-eight had either primary school or no formal education. The respondents were distributed across 155 towns across the six geopolitical zones of Nigeria, but most of them are from the southwest zone.

**Table 1:** Respondents’ Demographics

Item	Frequency (%)
Gender (n=1090)	
Female	423 (28.8%)
Male	667 (61.2%)
Age Group (n=809)	
18-25	67 (8.3%)
26-35	228 (28.2%)
36-45	211 (26.1%)
46-55	203 (25.1%)
55+	100 (12.4%)
Highest Education Status (n=1089)	
None	73 (6.7%)
Primary	7 (0.6%)
Secondary	19 (1.7%)
Diploma	38 (3.5%)
University/Polytechnic	458 (42.1%)
Postgraduate	494 (45.4%)
Geopolitical Zone	
Southwest	643 (60%)
North Central	212 (19.8%)
South-South	94 (8.8%)
South East	50 (4.7%)
North West	45 (4.2%
North East	27 (2.5%)

### Sources of information on the pandemic to the respondents

Majority of the respondents (34.4%) first became aware of the pandemic through mass media (TV/radio/newspaper), followed by the social media (21.8%) while 34.3% could not identify their first sources of information, but believed it was a combination of mass media and social media (Table 2).

**Table 2:** Educational Status versus sources of COVID-19 information

Educational Status	Mass media	Social media	From others	Hospital workers	Multiple sources	Total
Informal	61 (83.6%)	0	10 (13.7%)	0	2 (2.7%)	73 (100%)
Lowest	12 (46.2%)	5 (19.2%)	2 (7.7%)	0	7 (26.9%)	26 (100%)
Middle	20 (27.0%)	23 (31.1%)	2 (2.7%)	0	29 (39.2%)	74 (100%)
Highest	297 (32.2%)	215 (23.3%	0	10 (1.1%)	399 (43.3%)	921 (100%)
Total	438 (40.0%)	246 (22.5%)	14 (1.3%)	10 (0.9%)	386 (35.3%)	1094 (100%)

P<0.001

### Knowledge of COVID-19

Only three (0.6%) of the 1135 respondents are not aware of COVID-19 outbreak, and the three had no formal education. The vast majority of the respondents (81.4%) rated their knowledge of COVID-19 as good/very good, a further 17.1% rated it as average, while only 1.5% rated their knowledge of COVID-19 poor.

**Figure 1:**
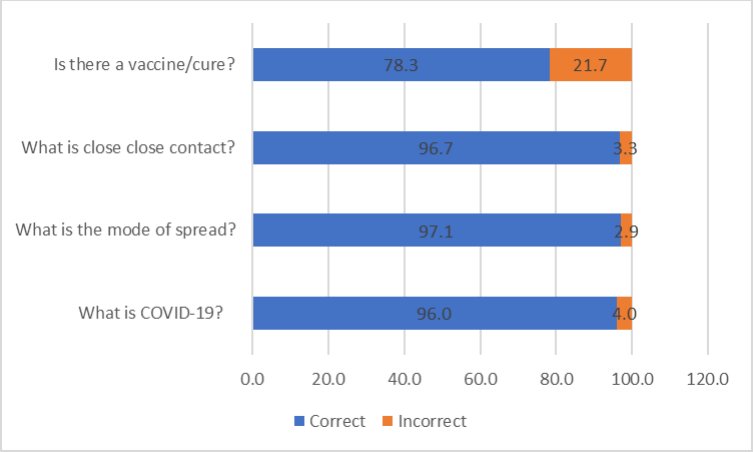
Knowledge of COVID-19

**Figure 2:**
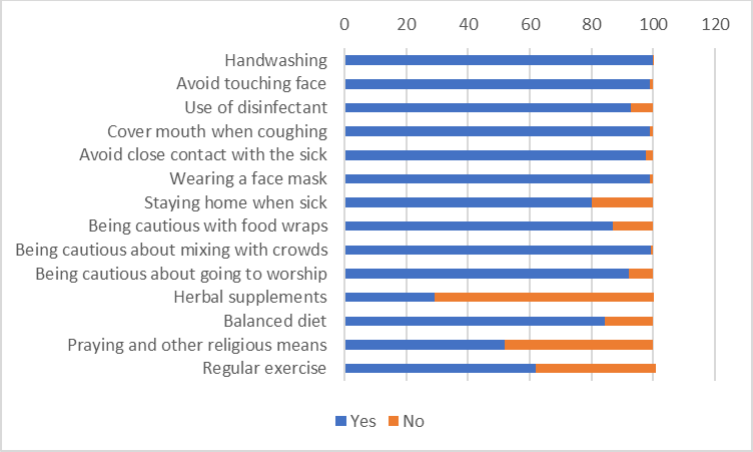
Knowledge of preventive measures of COVID-19.

Over 90% of the respondents had the correct knowledge of the cause and transmission of COVID-19; however, their knowledge of whether the disease has a cure or vaccine is poor (Figure 1). Figure 2 shows that the knowledge of the respondents regarding preventive measures are very good, but a good proportion of them think that general healthy behaviour such as balanced diets and regular exercise can prevent the disease. Close to 50% of the recipients believe in the efficacy of prayers in preventing the disease. As shown on Table 3, those with little or no education are more likely to believe in the efficacy of unproven preventive measures than those who are educated (P>0.001). However, their educational status had no significant association with the attitudes of respondents to the efficacy of hand washing and face mask in preventing the spread of COVID-19 (P>0.05).

**Table 3:** Comparison of respondents’ educational status with selected knowledge variables

Knowledge variable / Educational category	Yes	No	Not sure	Total	Statistical test	p-Value
*Prayer*					47.98	0.001
Lowest	69 (87.3%)	8 (10.1%)	2 (2.5%)	79		
Middle	43 (59.7%)	24 (33.3%)	5 (6.9%)	72		
Highest	415 (48.2%)	316 (36.7%)	130 (15.1%)	1012		
*Exercise*					21.72	0.001
Lowest	74 (85.1%)	10 (11.5%)	3 (3.4%)	87		
Middle	40 (56.3%)	22 (31.0%)	9 (12.7%)	71		
Highest	529 (60.4%)	243 (27.7%)	104 (11.9%)	876		
*Hand washing*					11.68	0.828
Lowest	99 (100%)	0	0	99		
Middle	74 (100%)	0	0	74		
Highest	1089 (99.8%)	2 (0.2%)	0	1091		
*Face Mask*					5.30	0.187
Lowest	94 (97.8%)	0	2 (2.2%)	96		
Middle	70 (98.6%)	1 (2.4%)	0	71		
Highest	908 (99.1%)	5 (0.5%)	4 (0.4%)	1084		

### Risk perception

**Figure 3:**
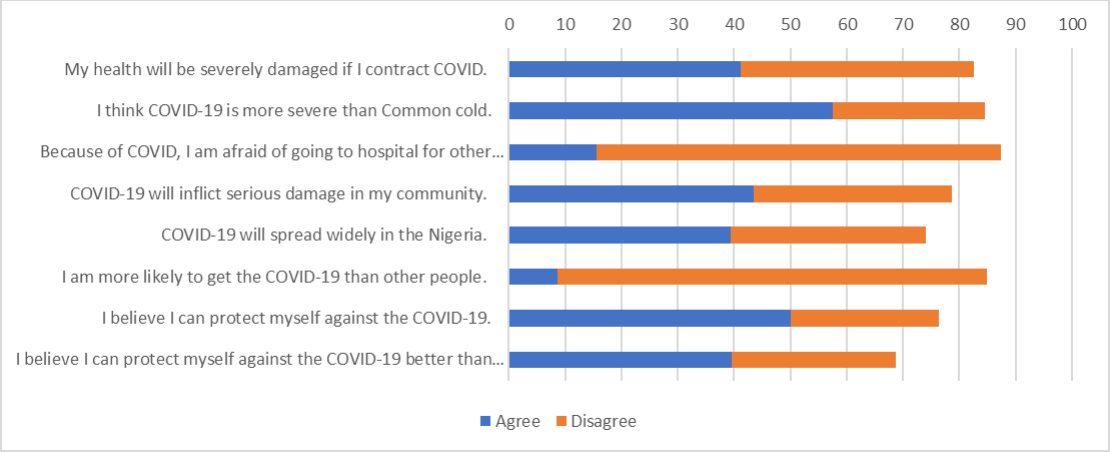
Risk perception of COVID-19

The risk perception of the participants is low to moderate (Figure 3). In particular, respondents did not feel that they had to limit their visitation to the hospitals, even when sick with other illnesses. Most of them do not believe that they are more likely to get the disease

### Preventive measure taken

**Figure 4:**
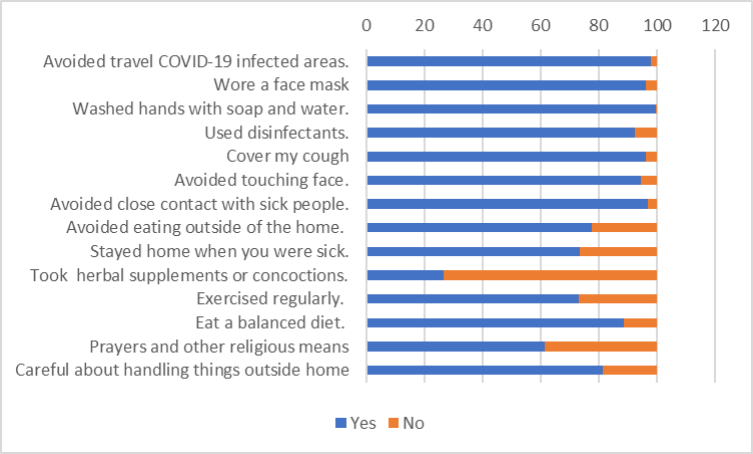
Preventive measures that were taken against COVID-19

A large percentage of the respondents are compliant with commonly advised COVID-19 preventive measures such face mask usage, hand hygiene and social distancing (Figure 4). However, close to three-quarters of the respondents have also taken to non-orthodox anti-COVID-19 measures such as herbal supplement, praying and exercise.

### Concerns and Trust in Institutions about COVID-19

**Figure 5:**
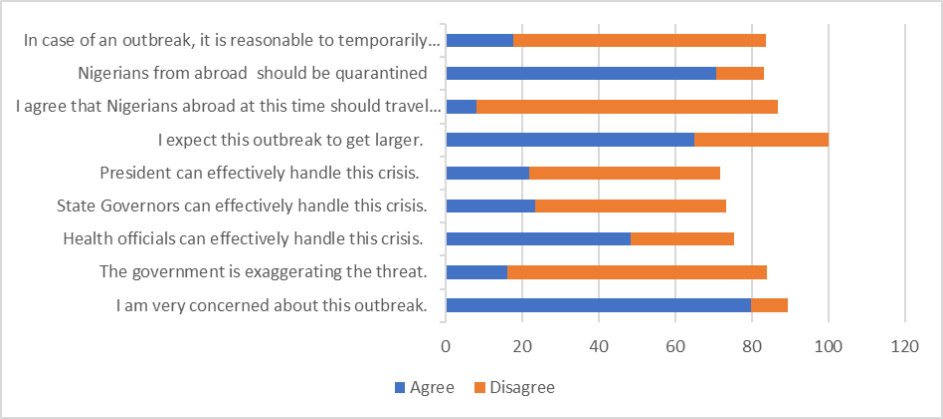
Concerns about COVID-19

Most of the respondents worry about the pandemic, and they do not feel the government is exaggerating the issue (Figure 5). However, while about 50% trust health officers to handle the crisis well, less than 25% of them trust the Federal and State governments to handle the situation well. Most believe that persons arriving from abroad should be quarantined and that no group of people should be discriminated against because of the disease. Less than half of the respondents trust the government or any of its parastatals to handle the pandemic effectively.

## Discussion

Majority of the respondents in this study were well aware of COVID-19, being a new disease that has within a short time infected about one in every 250 persons in the world (Dong, Du and Gardner, 2020; Center for Systems Science and Engineering (CSSE) at Johns Hopkins University (JHU)). The disease, which was declared a pandemic in March 2020 had no vaccine and cure at the time. Since the disease is transmitted by air and fomites and has R_0_ greater than 1, it means that the most important public health approach to controlling the spread of the disease is by preventing person-to-person transmission. The three most important approach to preventing person-to-person transmission is by wearing masks, observing hand hygiene and social distancing. However, studies from across the world have shown that adherence to these COVID-19 containment measures differ across the world and are influenced by several factors including knowledge, attitude and risk perception (Cori *et al.*, 2020).

Both the awareness and knowledge about COVID-19 are generally high among respondents in the present study. But there also exist some knowledge gap as many believe that general good healthy behaviours such as balanced diets and regular exercise are protective against COVID-19. Similarly, about half of the respondents believe that prayers can prevent the disease. This is not surprising because a lot of them got their information about the disease from the social media. The social media are notorious for disseminating fake news and information (Cinelli *et al.*, 2020; O’Connor and Murphy, 2020). Even the mass media are not free from this because some main-stream journalists get their information from the social media (Ireton, Posetti, and UNESCO, 2018). Those who have little or no education are most likely to get their knowledge of COVID-19 from the mass media. The proportion of those who cannot read and understand English in Nigeria is about 43% (National Bureau of Statistics). These people depend on the mass media or other people (family members and neighbours) to stay informed; although, only 12.5% of respondents in this study gotCOVID-19 related information from other people. The mass media are no doubt crucial if the government wants to keep the less educated informed about COVID-19. The government and other stake holders must have programmes on radios and television in both local languages and English which are dedicated to informing the public about the pandemic (O’Connor and Murphy, 2020). These programmes will counter fake news about the pandemic and provide alternative and free source of COVID-19 related information (Haciyakupoglu *et al.*, 2018; Berduygina, Vladimirova and Chernyaeva, 2019).

Surprisingly, just 0.9% of the respondents got their first information about the pandemic from healthcare workers. Interestingly none of those with the lowest education got their information from healthcare workers. In the days before the mass media and social media, healthcare workers were valued sources of health-related information (Hesse *et al.*, 2005). Studies have also shown that information from healthcare workers is often more accurate than from most other sources (Hesse *et al.*, 2005). Therefore, the decline in the proportion of medical related information from healthcare workers coupled with the simultaneous rise in the proportion due to the social media can only result in a drop in the quality of health information received by the people. As reflected in Table 3, less educated respondents who mostly relied on a single source of information to learn about the disease are more likely to believe in the efficacy of unproven preventive measures.

When compared to their knowledge, the risk perception of the respondents is low. This notwithstanding, most of the respondents were worried about the disease. Over 60% expected the outbreak to get larger in the country and they also want persons arriving from travels overseas to be quarantined/ isolated. Perhaps this explains the high incidence of preventive measures that the study participants have taken in the past.

The present study showed that respondents believed healthcare workers are capable of managing the pandemic. On the other hand, information and directives from the Nigerian Government on the pandemic was not well received by the respondents. This finding is similar to studies from other countries (Askarian, Aramesh and Palenik, 2006; Saqlain *et al.*, 2020). Paradoxically, most believe that the government is not exaggerating the figures, yet less than one-fifth of the respondents trust the federal and state governments to handle the pandemic well. This might be the result of the long-held belief that governments in Nigeria are corrupt. This is like the situation in most low-income countries where there is little trust between the government and the people (Chakraborty and Maity, 2020; Hopman, Allegranzi and Mehtar, 2020).

### Limitations

The study has the usual limitations of online surveys. It is based on convenience sampling and those who are poorly educated and those who have limited access to the internet are poorly represented. Therefore, the study may not truly represent the adult population of Nigeria. To address this limitation, an interviewer administered purposive sampling of respondents with little or no education was conducted.

## Conclusion

Awareness about COVID-19 was generally high in the present study, but there exist some important knowledge gaps with particular emphasis on protective measures against the disease. The belief in non-proven or non-orthodox means such as balanced diets, regular exercise and prayers as protective against COVID-19 needs correction.

The risk perception of COVID-19 is also not encouraging just as low level of confidence was demonstrated against government capability to manage the epidemics. There is thus need for intensified advocacy and proper public orientation to curb further spread of COVD-19 in our setting.

### Conflict of Interest:

All the authors declare no conflict of interest.

**List of Abbreviations:**
SARS-COV-2-: Severe acute respiratory syndrome-corona virus
WHO -: World Health Organization
