# KNOWLEDGE, ATTITUDE AND RISK PERCEPTION OF COVID-19 AMONG NIGERIANS

**Published:** 2022-08-17

**Authors:** Fadeyi Abayomi, Adeoti Adekunle Olatayo, Adeboye Muhammed Akanbi Nurudeen, Awosanya Joseph Abioye, Oluwadiya Ibironke Omowumi, Oluwadiya Kehinde Sunday

**Affiliations:** 1 Medical Microbiology & Parasitology Department, University of Ilorin, Ilorin, Kwara State, Nigeria; 2 Internal Medicine Department, Ekiti State University, Ado-Ekiti, Ekiti State, Nigeria; 3 Paediatric & Child Health Department, University of Ilorin, Ilorin, Kwara State, Nigeria; 4 Medical Microbiology &Parasitology Department, Ekiti State University, Ado-Ekiti, Ekiti State, Nigeria; 5 Demography & Social Statistics Department, Obafemi Awolowo University, Ile Ife, Osun State, Nigeria; 6 Orthopedic Unit, Surgery Department, Ekiti State University, Ado-Ekiti, Ekiti State, Nigeria

In the version of this article initially published, there were errors resulting from merged authors’ names. The following sentences highlight the errors and the corrected details:

^1^ Abayomi Fadeyi, ^2^ Adekunle Olatayo Adeoti, ^3^ Muhammed Akanbi Nurudeen Adeboye, ^4^ Joseph Abioye Awosanya, ^5^ Ibironke Omowumi Oluwadiya, ^6*^ Kehinde Sunday Oluwadiya.

**Should read:**

^1^Fadeyi Abayomi, ^2^ Adeoti Adekunle Olatayo, ^3^Adeboye Muhammed Akanbi Nurudeen, ^4^Awosanya Joseph Abioye, ^5^Oluwadiya Ibironke Omowumi, ^6*^Oluwadiya Kehinde Sunday.

